# Iatrogenic injury of the portal vein at the time of surgical excision of giant gastrointestinal stromal tumor and our surgical procedure

**DOI:** 10.1186/1749-8090-8-S1-P61

**Published:** 2013-09-11

**Authors:** K Ergunes, H Iner, F Tatar, I Yurekli, A Gurbuz

**Affiliations:** 1 Izmir Katip Celebi University Ataturk Training and Research Hospital, Department of Cardiovascular Surgery, Izmir, Turkey; 2 Izmir Katip Celebi University Ataturk Training and Research Hospital, Department of General Surgery, Izmir, Turkey

## Background

Gastrointestinal stromal tumor is common in the gastrointestinal tract. Surgical excision of gastrointestinal stromal tumor is extremely important to avoid portal vein injury. Iatrogenic ınjury of portal vein is rare but devastating.

## Method

The patient was 45-year-old female. Abdominal tomography showed a tumor in 10 cm diameter adjacent to the duodenum and head of pancreas.

## Results

The portal and superior mesenteric vein injuries developed at the time of tumor excision by general surgeons. We joined the operation of general surgeons. Proximal and distal region of the portal and superior mesenteric vein were compressed with hand to stop bleeding. The portal and superior mesenteric veins were repaired with 6/0 prolene suture. The portal and superior mesenteric vein were dissected from tumor. The tumor was extracted. Histopatologic examination of tumor showed gastrointestinal stromal tumor. The patient was performed partial gastrectomy, cholecystectomy, pancreaticoduodenectomy by general surgeons. She was discharged on day 24.

## Conclusion

Urgent surgical treatment of iatrogenic injury of the portal and superior mesenteric vein is important to prevent mortality.

